# Can Neurostimulation Prevent the Risk of Alzheimer's Disease in Elderly Individuals with Schizophrenia?

**DOI:** 10.3389/fpsyt.2013.00111

**Published:** 2013-09-18

**Authors:** Adonis Sfera

**Affiliations:** ^1^Patton State HospitalPatton, CA, USA; ^2^South Coast Clinical TrialsAnaheim, CA, USA

**Keywords:** amyloid markers, tau pathology, neuroprotection, antipsychotic medications, beta secretase, neurostimulation

## Introduction

As patients with schizophrenia live longer, because of better medical management, suicide prevention, and appropriate nutrition, we are beginning to face a new phenomenon: elderly patients with chronic schizophrenia developing Alzheimer's disease.

In the past two decades several neurostimulation methods have been introduced in psychiatry. These include transcranial magnetic stimulation (TMS), vagus nerve stimulation (VNS), deep brain stimulation (DBS), and transcranial direct current stimulation (tDCS). To date only TMS and tDCS have been used in the treatment of positive, negative, and cognitive symptoms of schizophrenia (1).

These treatment modalities show promise to decrease the risk of Alzheimer's disease in elderly individuals with schizophrenia, but in order to optimize their benefits it is essential to recognize the differences in the cognitive pathology involved.

Since it is possible that specific neurostimulatory modalities or sites of stimulation affect different regions of the brain or domains of neurocognitive pathology in a different manner, it is necessary to have a better understanding of the cognitive deficits in each condition.

## Similarities and Differences between Domains of Cognitive Pathology

In many ways schizophrenia can be considered as a disease of accelerating aging. It presents with cognitive deficits and metabolic abnormalities. Schizophrenia is associated with an increase in mortality, resulting in a decrease in average life span of 20% (2).

Patients with schizophrenia show a rapid cognitive decline after the age of 65 in addition to the already existing cognitive deficit. Clinical studies suggest that severe cognitive impairment is common among elderly patients with schizophrenia who reside in long-stay psychiatric institutions (3).

In spite of this data, Alzheimer's dementia *per se* is diagnosed in only 9% while other dementing diseases are diagnosed in 4% of the individuals with schizophrenia (4). This finding has led some to the assumption that these patients are somehow resistant to Alzheimer's disease.

Despite neurocognitive similarities between patients with Alzheimer's dementia and schizophrenia, there are also major neuropsychological as well as laboratory differences. In general biological markers show a lack of increased Alzheimer's pathology in patients with schizophrenia, while neuropsychological testing show differences in the affected domains of cognitive deficits (5).

### Neuropsychological differences

The range of cognitive impairments in individuals with schizophrenia differs from the one in Alzheimer's disease. In elderly schizophrenic individuals there is less forgetfulness and delayed recall. Most deficits are seen in the processing speed, episodic memory, working memory, and executive function (6). Elderly individuals with schizophrenia scored lower on digit symbol substitution, picture completion, and block design in Wechsler Adult Intelligence Scale Revised (WISE-R) as compared to Alzheimer's disease patients (6).

### Differences in biological markers

In post mortem studies, the degree of senile plaques or neurofibrillary tangles was not different in the schizophrenia group compared with non-schizophrenic psychiatric disorders (5).

#### Amyloid markers

In spite of cognitive deficits, most studies failed to find increased amyloid deposits in patients with schizophrenia, even after age 65, leading to the belief, by some, that patients with schizophrenia are protected from developing Alzheimer's disease (6).

#### Tau pathology markers

Since a more pronounced cognitive decline has been described in older schizophrenic patients, it has been hypothesized that these patients might be at a higher risk of developing AD. In spite of this belief, no significant differences in CSF total tau and phospho-tau levels were found in patients with schizophrenia and controls (7).

#### Functional MRI

Just like different memory domains are impaired in schizophrenia vs. Alzheimer's disease, functional MRI shows different areas of brain tissue loss in the two conditions. Areas with specifically low gray matter volume in AD were distributed within the neocortical associative areas, while in elderly schizophrenia patients these were confined to the posterior part of the anterior cingulate gyrus (5). Other *in vivo* neuroimaging studies have provided evidence of decreases in the gray matter volume of the cingulate gyrus in subjects with schizophrenia as compared to healthy controls (8).

## Is Neurostimulation Neuroprotective?

Neuroprotection is a new direction in the treatment of disorders that lead to neurodegeneration as a result of deterioration of neurons due to excitotoxicity, apoptosis, oxidative stress. The concept of neuroprotection has implications for mood and cognitive disorders such as schizophrenia (9).

Recent studies suggest that neurostimulation may have neuroprotective effects (10). For example it is well known that DBS of subthalamic nucleus, frequently used in treating Parkinson's disease, was also found to be neuroprotective in rats (11).

Deep TMS used primarily for the treatment of major depressive disorder, was found to have positive effects on working memory if applied over the left prefrontal cortex (12). In this respect it was found beneficial for use in negative and cognitive symptoms of schizophrenia (13).

Various psychotropic agents used in the treatment of schizophrenia and dementia are neuroprotective as well, including drugs such as: clozapine (9), quetiapine (14), nicotinic agonists, especially alpha 7 ligands (15), lithium (16, 17), and selective serotonin uptake inhibitors (SSRI) (9). Using neurostimulation as an adjunctive therapeutic technique might augment the effect of psychotropic drugs by offering extra benefit, especially in poor responders.

In this respect a recent study showed that tDCS provided an alternative and potentially superior approach to the treatment of psychotic symptoms in medication-resistant schizophrenia (18).

Several studies (19–23) showed that improvement of cognitive functions was promoted by anodal tDCS stimulation. For example, stimulation over the left DLPFC has been shown to improve executive abilities and working memory performance in individuals with dementia (24).

Since gray matter loss in the elderly schizophrenia patients was noted in the posterior part of the anterior cingulate gyrus, it would be interesting to look at tDCS anodal stimulation in that particular area in order to prevent development of Alzheimer's dementia in this group.

## Conclusion

Elderly individuals with schizophrenia present with an accelerating cognitive decline. In this respect it is considered as a disease of premature aging. DBS was found to be beneficial in neurodegenerative disorders such as Parkinson's disease and Alzheimer's disease. TMS has shown benefit in improving working memory and cognitive symptoms in schizophrenia. tDCS was found to be beneficial in positive, negative, and cognitive symptoms of schizophrenia. tDCS stimulation over the left DLPFC has been shown to improve executive abilities and working memory in individuals with dementia. Overall, neurostimulation in schizophrenia may be neuroprotective and prevent the risk of Alzheimer's disease.
